# Operative and nonoperative treatment of clavicle fractures in adults: A systematic review of 1,190 patients from the literature

**DOI:** 10.3109/17453674.2011.652884

**Published:** 2012-02-08

**Authors:** Kaisa J Virtanen, Antti O V Malmivaara, Ville M Remes, Mika P Paavola

**Affiliations:** ^1^Department of Orthopaedics and Traumatology, Helsinki University Central Hospital; ^2^Centre for Health and Social Economics, Institute of Health and Welfare, Helsinki, Finland

## Abstract

**Background and purpose:**

Traditionally, clavicle fractures have been treated nonoperatively. However, many recent studies have concentrated on the results of operative treatment. We assessed and compared the outcomes of operative and nonoperative treatment for acute clavicle fractures in adults.

**Methods:**

We performed a systematic search of the medical literature from 1966 until the end of March 2011. We included randomized controlled trials and controlled clinical trials comparing operative and nonoperative treatment and studies comparing different operative and nonoperative treatments. We required that there should be at least 30 adult patients and a follow-up of at least 6 months in each individual trial. We used the GRADE method to assess the quality of evidence.

**Results:**

6 randomized controlled trials (n = 631) and 7 controlled clinical trials (n = 559) were included. There was moderate-quality evidence (i.e. of grade B) (1) that surgery has considerable effectiveness on better function and less disability at short follow-up, (2) of similar risk of relatively mild complications after operative or nonoperative treatment, (3) that delayed union and nonunion were more common in patients who were treated nonoperatively than in those treated operatively, and (4) that the osteosynthesis method had no effect on the incidence of delayed union or nonunion. Only 1 controlled clinical trial was found on lateral clavicle fractures with very limited (grade D) evidence.

**Interpretation:**

Patients treated operatively have slightly better function and less disability than those treated nonoperatively at short follow-up, but then the effectiveness diminishes and is weak at 6 months. The different operative techniques may not differ in effectiveness or in adverse effects, but the evidence is very limited or conflicting. Surgery could be considered for active patients who require recovery to the previous level of activity in the shortest possible time.

Clavicle fractures comprise 2% of all fractures and 35–45% of all shoulder girdle injuries in adults (Nordqvist and Petersson 1994, Postacchini et al. 2002). The incidence in western countries is around 50–64 per 10^5^ (Nordqvist and Petersson 1994, Nowak et al. 2000). They are more common in men (68%) (Postacchini et al. 2002).

Clavicle fractures are classified according to the anatomical site and degree of displacement (Neer 1960, Allman 1967, Robinson 1998). Most clavicle fractures are situated in the middle part (81%), whereas lateral (17%) and medial fractures (2%) are much less common (Postacchini et al. 2002).

By tradition, midshaft clavicle fractures have been treated nonoperatively with the arm immobilized in a sling for few weeks. The goal is to restore function of the upper extremity and to prevent any constant disability from the injury. Recently, there has been increasing interest in the operative treatment (COTS 2007, Smekal et al. 2009).

In this systematic review, based on randomized controlled trials and controlled clinical trials, we assessed the effectiveness and adverse effects of operative and nonoperative treatment of acute clavicle fractures in adults.

## Materials and methods

Methods for the inclusion and exclusion criteria, data extraction, and data synthesis were specified in advance and documented in a protocol.

### Literature search

An information specialist made an electronic database search of the literature without language restrictions using CDSR, DARE, CCTR, CINAHL, Ovid MEDLINE In-Process & Other Non-indexed Citations, Ovid MEDLINE, Journals@Ovid, Current Controlled Trials Register, and EMBASE from 1966 until the end of March 2011. We used the following terms to search all trial registers and databases: fractures, fracture fixation, fracture healing, clavicle, and collar bone (Appendix, see supplementary data). The latest search was run on March 31, 2011. Detailed search strategy is available from the authors. In addition, we contacted the study authors by e-mail to obtain more detailed information of studies.

### Inclusion and exclusion criteria

Eligible studies were randomized controlled trials and controlled clinical trials comparing operative with nonoperative treatment, operative treatment with another operative treatment, and nonoperative treatment with another nonoperative treatment for acute clavicle fractures. Studies had to involve at least 30 adult patients (≥ 18 years of age). The minimum follow-up time was 6 months. Our primary outcome measures were functional assessments: Constant shoulder score (CS), Disabilities of the Arm, Shoulder and Hand Score (DASH), and visual analog scale for pain (VAS). Secondary outcome measures were fracture union, range of motion (ROM), return to previous activity, and complications. We excluded studies dealing with non-acute fractures (treatment after 3 weeks) and 3 studies written in Chinese.

Study selection and assessment of methodological quality were done by 3 independent investigators (KV, MP, and VR). Discrepancy between investigators was solved by negotiation or, when necessary, by a fourth investigator (AM). We started with all the abstracts identified and excluded those that did not discuss the subject. From full text articles, we excluded retrospective trials and other studies that did not fulfill the eligibility criteria.

### Data extraction and quality assessment

Data extraction from each study included was done by KV with a predetermined data extraction form. MP checked the extracted data. The data extraction form consisted of 3 sheets: (1) characteristics of the studies (author, study design, fracture location, fracture classification, fracture displacement, intervention, follow-up time, number of patients, and percentage of dropouts), (2) criteria for risk of bias, and (3) effectiveness of the study (primary and secondary outcome measures, complications, union, and nonunion). On assessing complications, we used recommendations of the Cochrane Handbook of Systematic Reviews of Interventions (Loke et al. 2006). As suggested, we documented all reported complications in order to have wide coverage of the adverse effects. Assessment of risk of bias in trials was performed according to Furlan et al. (2009). We assessed the trials to have a low risk of bias if at least 6 out of 12 criteria were met. If the trials met less than 6 criteria, we rated the risk of bias as high.

### Data synthesis

Due to the clinical heterogeneity in patient populations, treatments, outcomes, and fracture morphology, we could not pool the effect sizes in a meta-analysis. Instead, we summarized findings by strength of evidence. We used the difference in means as a summary measure. The overall quality was assessed by 3 authors (KV, MP, and AM). The evidence for each outcome was evaluated by using the GRADE approach, as recommended by the Cochrane Back Review Group (Furlan et al. 2009). The quality of the evidence on a specific outcome is based on 5 domains: (1) limitations of the study design, (2) inconsistency, (3) indirectness, (4) imprecision of results (insufficient or imprecise data), and (5) publication bias across all studies measuring that particular outcome. The quality starts at high when at least 2 high-quality RCTs provide results for the outcome, and is reduced by 1 level for each of the domains not met. The following criteria were used for assessment of the quality of evidence:

High-quality evidence = there are consistent findings in at least 75% of RCTs with no limitations of the study design, consistency, directness, or precision and no known or suspected publication bias. Moderate-quality evidence = 1 of the domains is not met. Low-quality evidence = 2 of the domains are not met. Very low-quality evidence = 3 of the domains are not met. No evidence = no RCTs were identified that addressed this outcome.

We considered a minimal clinically important difference in pain on the VAS scale to be more than 20 units (Tubach et al. 2006), and in disability more than 10 units of the DASH score (Gummesson et al. 2003, Roy et al. 2009).

## Results

After elimination of duplicates, we found 1,072 abstracts from the electronic database searches reporting clavicle fractures. Most of the excluded studies did not address clavicle fractures or did not fulfill the inclusion criteria. For the thorough examination, we accepted 230 publications. From these studies, we judged 27 to be potentially appropriate. For the systematic review, 14 studies qualified, 6 of which were randomized controlled trials and 8 of which were controlled clinical trials (Figure). We found 2 studies originating from the same patient population, and we have therefore only reported results from the more recent study (Smekal et al. 2009, 2011). Studies originated from several countries: Austria (Smekal et al. 2009, 2011), Canada (COTS 2007), China (Shen et al. 2008), Germany (Jubel et al. 2005, Bohme et al. 2011), India (Kulshrestha et al. 2011), the Netherlands (Hoofwijk and van der Werken 1988), Taiwan (Lee et al. 2007, 2008, Pai et al. 2009, Hsu et al. 2010), the United Kingdom (Ferran et al. 2010), and the United States (Judd et al. 2009). Only 1 study discussed lateral clavicle fractures (Hsu et al. 2010). We excluded 3 studies, since they were written in Chinese.

**Figure.**
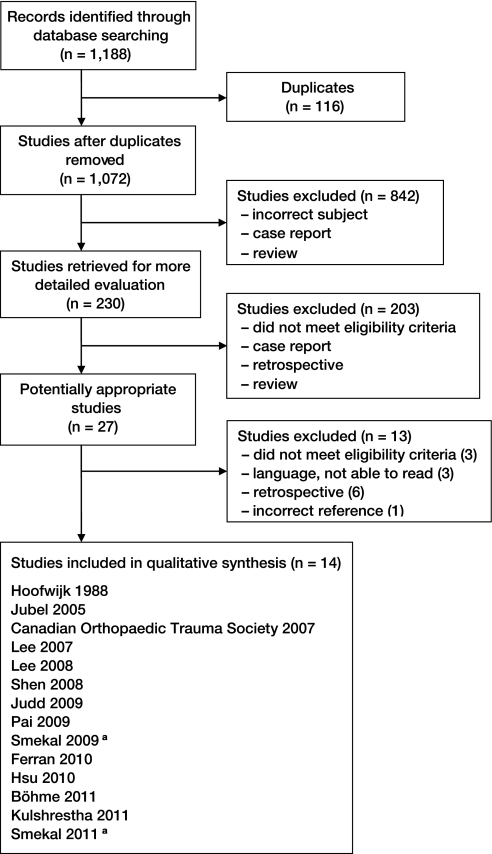
Flow chart illustrating number of trials evaluated at each stage in the systematic review of clavicle fractures. **^a^** Studies are assessed to originate from the same patient population. Results are reported from the recent (2011) study, thus the final number of studies was 13.

Table 1 lists study designs, patients, follow-up times, fracture types, interventions, and control treatments in the studies. The 13 studies involved 1,190 patients. There were 6 randomized controlled trials (631 patients) (Hoofwijk and van der Werken 1988, COTS 2007, Shen et al. 2008, Judd et al. 2009, Ferran et al. 2010, Smekal et al. 2011) and 7 controlled clinical trials (559 patients) (Jubel et al. 2005, Lee et al. 2007, Lee et al. 2008, Pai et al. 2009, Hsu et al. 2010, Bohme et al. 2011, Kulshrestha et al. 2011). Sample sizes differed considerably (32–157 patients) between the studies. Follow-up times in the studies were long enough to evaluate the effect of the treatment after clavicle fracture (6–30 months).

**Table 1.** Characteristics of the included studies (n=13) in systematic review of clavicle fractures

A	B	C	D	E	F	G	H	I	J
Hoofwijk 1988 Netherlands	RCT	M	NM	< / >1 clavicle width	Rucksack bandage (78)	Mitella (79)	10 months	157/152	3.2
COTS 2007 Canada	RCT	M	NM	Completely displaced, no cortical contact between main fragments	Plate (67)	Sling (65)	1 year	132/111	15.9
Shen 2008 China	RCT	M	NM	Compeletely displaced	3D reconstruction plate (67)	Superior reconstruction plate (66)	1 year	133/117	12.0
Judd 2009 United States	RCT	M	NM	Displaced or angulated	Hagie pin (29)	Sling (28)	1 year	57/50	12.3
Smekal 2009, 2011 Austria **^b^**	RCT	M	OTA	Displaced, no cortical contact between main fragments	ESIN (60)	Sling (60)	2 years	120/112	6.7
Ferran 2010 UK	RCT	M	NM	Displaced and shortened with complete overlap of bone ends	Rockwood pin (17)	Plate (15)	1 year	32/32	0
Jubel 2005 Germany	CCT	M	Allman OTA	Allman I OTA A or B	ESIN (26)	Rucksack bandage (27)	6 months	53/53	0
Lee 2007 Taiwan	CCT	M	NM	Not mentioned	Knowles pin (32) **^a^**	Dynamic compression plate (30) **^a^**	30 months	69/62	10.1
Lee 2008 Taiwan	CCT	M	NM	Not mentioned	Knowles pin (56) **^a^**	Plate (32) **^a^**	1 year	103/88	14.6
Pai 2009 Taiwan	CCT	M	NM	Not mentioned	Locking compression plate (29) **^a^**	Nonlocking plate (35) **^a^**	1 year	76/64	15.8
Hsu 2010 Taiwan	CCT	L	Neer	Neer II	Hook plate (35)	Tension band wire (30)	6 months	65/65	0
Böhme 2011 Germany	CCT	M	AO	AO 15-B: types A1, A2, B1, B2, C1, C2	Plate (53) ESIN (20)	Rucksack bandage (47)	8 months	120/96	20
Kulshrestha 2011, India	CCT	M	OTA Robinson	OTA 15-B Robinson 2B1 and 2B2	Plate (45)	Sling (28)	18 months	73/68	6.8

A Author, year, country COTS = Canadian Orthopaedic Trauma Society B Study design RCT randomized controlled trial CCT controlled clinical trial C Location of the fracture M middle L lateral D Classification of the fracture NM not mentioned E Displacement of the fracture OTA Orthopaedic Trauma Association F Intervention (no. of patients) ESIN elastic stable intramedullary nail G Control (no. of patients) H Follow-up time I No. of patients at initiation / at follow-up J Percentage of drop-out

**^a^** Studies report no. of patients only at follow-up.

**^b^** Studies are assessed to originate from the same patient population. Results are reported from the recent (2011) study.

Table 2 summarizes the methodological quality of the trials according to Furlan et al. (2009). Of 13 studies, 2 were considered to have a high risk of bias (Bohme et al. 2011, Smekal et al. 2011).

**Table 2.** Criteria for risk of bias assessment for the trials included (n=13) in the systematic review (Furlan et al. 2009). If ≥ 6 criteria were met, the trial is assessed to have low risk of bias

Author, year, country	1.	2.	3.	4.	5.	6.	7.	8.	9.	10.	11.	12.	Total
Randomized controlled trials													
Hoofwijk 1988 Netherlands	Yes	Unclear	NA	NA	NA	No	Yes	Yes	Yes	Yes	Yes	Unclear	6
COTS 2007 Canada	Yes	Yes	NA	NA	NA	No	Yes	Yes	Yes	Yes	Yes	Yes	8
Shen 2008 China	Yes	Yes	Unclear	Unclear	Yes	No	Unclear	Yes	Yes	Yes	Yes	Yes	8
Judd 2009 United States	Yes	Yes	NA	NA	NA	No	Unclear	Yes	Yes	Yes	Yes	Yes	7
Smekal 2009, 2011 Austria **^a^**	No	Unclear	NA	NA	NA	No	No	Yes	Yes	Yes	Yes	Yes	5
Ferran 2010 United Kingdom	Yes	Yes	NA	NA	NA	Unclear	Yes	Yes	Yes	Yes	Yes	Unclear	7
Controlled clinical trials													
Jubel 2005 Germany	No	No	NA	NA	NA	Yes	Unclear	Yes	Yes	Yes	Yes	Yes	6
Lee 2007 Taiwan	No	No	NA	NA	NA	Yes	Yes	Yes	Yes	Yes	Yes	Unclear	6
Lee 2008 Taiwan	No	No	NA	NA	NA	Yes	Yes	Yes	Yes	Yes	Yes	Yes	7
Pai 2009 Taiwan	No	No	No	NA	NA	Yes	Unclear	Yes	Yes	Yes	Yes	Yes	6
Hsu 2010 Taiwan	No	No	NA	NA	NA	Yes	Yes	Yes	Yes	Yes	Yes	Yes	7
Böhme 2011 Germany	No	No	NA	NA	NA	No	Unclear	Yes	No	Yes	Yes	No	3
Kulshrestha 2011 India	No	No	No	NA	NA	Yes	Yes	Yes	Yes	Yes	Yes	Yes	7

1. Was the method of randomisation adequate? 2. Was the treatment allocation concealed? 3. Was the patient blinded to the intervention? 4. Was the care provider blinded to the intervention? 5. Was the outcome assessor blinded to the intervention? 6. Was the drop-out rate described and acceptable? 7. Were all randomized (CCT: allocated) participants analysed in the group they were allocated? 8. Are reports of the study free of suggestion of selective outcome reporting? 9. Were the groups similar at baseline regarding the most important prognostic indicators? 10. Were co-interventions avoided or similar? 11. Was the compliance acceptable in all groups? 12. Was the timing of the outcome assessment similar in all groups?

**^a^** Studies are assessed to originate from the same patient population. Results are reported from the recent (2011) study. Abbreviations: NA = Not applicable COTS = Canadian Orthopaedic Trauma Society

### Treatment of middle-third clavicle fractures

Of 6 randomized controlled trials, 3 compared operative treatment to nonoperative treatment (COTS 2007, Judd et al. 2009, Smekal et al. 2011), 2 compared different operative treatments (Shen et al. 2008, Ferran et al. 2010), and 1 compared two types of nonoperative treatment (Hoofwijk and van der Werken 1988). Of 6 controlled clinical trials, 3 compared operative treatment to nonoperative treatment (Jubel et al. 2005, Bohme et al. 2011, Kulshrestha et al. 2011) and 3 compared operative methods (Lee et al. 2007, 2008, Pai et al. 2009) (Table 1).

*Operative vs. nonoperative treatment.* In a 1-year follow-up of 132 fracture patients comparing plate osteosynthesis to sling, the values in CS were 96 vs. 90 (p < 0.01) and in DASH score 5 vs. 15 (p < 0.01). Mean time to radiographic union was 16 weeks after operative treatment and 28 weeks after nonoperative treatment (p = 0.001) (COTS 2007).

Smekal et al. (2011) compared elastic stable intramedullary nailing (ESIN) to sling in a 2-year follow-up study of 120 fracture patients. Mean time to union was 12 weeks in the ESIN group and 17 weeks in the sling group (p = 0.01). They found 4% clavicular shortening in the ESIN group and 5% shortening in the sling group. Mean DASH scores were 0.5 and 3 respectively (p = 0.03), and mean Constant scores were 98 and 95 (p = 0.02). Operative treatment secured union better than nonoperative treatment (p = 0.01).

When Hagie pin was compared to a sling, the values in single-assessment numeric evaluation (SANE) were 94 vs. 97 at 1-year follow-up (p > 0.05) and 96 vs. 98 (p > 0.05) in L’Insalata. 26/27 fractures in the operative group united and 22/23 fractures in the nonoperative group united (p > 0.05) (Judd et al. 2009).

At 6-month follow-up of 53 fracture patients comparing ESIN to rucksack bandage, mean CS was 98 vs. 90 (p < 0.001) and mean DASH score was 2 vs. 10 (p < 0.001). Mean VAS score was 1 vs. 14 (p < 0.05) (Jubel et al. 2005).

Böhme et al. (2011) compared rucksack bandage to operative treatment (ESIN or plate) in a controlled clinical study of 120 fracture patients. At 8-month follow-up, mean CS was 97 in the ESIN group, 94 in the plate group, and 90 in the rucksack bandage group (p = 0.01).

Kulshrestha et al. (2011) found a 6-point difference in CS in favor of surgery between patients treated with a sling or plate at 18-month follow-up (p < 0.0001). At 6-month follow-up, 45/45 fractures in the operative group were united as compared to 20/28 fractures in the nonoperative group (p = 0.002).

*Operative vs. operative treatment.* In a 4-month period, 63/67 fractures stabilized with a 3-dimensionally contoured plate had united compared to 43/66 fractures stabilized with a superior plate (p < 0.05) (Shen et al. 2008).

In patients treated with Rockwood pin (n = 17) or plate (n = 15), mean CS was 92 vs. 88 (p = 0.365) and mean Oxford shoulder score was 45 vs. 45 at the 1-year follow-up. All fractures healed in both treatment groups (Ferran et al. 2010).

When comparing a locking compression plate (n = 29) with nonlocking plate (n = 35) in elderly patients (60–83 years), union rate was 97% vs. 97%, CS was 92 vs. 89, and VAS was 2.1 vs. 2.2 at 1-year follow-up (Pai et al. 2009).

At 1-year follow-up, mean CS was 95 vs. 93 and mean union rate was 100% vs. 97% in patients treated with Knowles pin (n = 56) or plate (n = 32) (Lee et al. 2008). In another study comparing Knowles pin (n = 32) and plate (n = 30), mean CS was 85 vs. 84 and union rate was 100% vs. 97% at the 30-month follow-up (Lee et al. 2007).

*Nonoperative vs. nonoperative treatment.* At 10-month follow-up comparing mitella (n = 79) to rucksack bandage (n = 78), mean clinical consolidation time was 3.6 vs. 3.8 weeks, and mean VAS was 1.8 vs. 2.6 (Hoofwijk and van der Werken 1988).

*Complications.*Some authors reported a variety of complications, whereas some reported them very briefly (Table 4). Risk to nonunion in patients treated nonoperatively varied from 0% to 29%. Patients treated operatively had notably lower risk to nonunion (0–4%). The operative method (plate, ESIN, Knowles pin, or Hagie pin) had no influence on the nonunion rate. Risk of wound infection was low (0–5%) after operative treatment, except in 2 studies. Judd et al. (2009) noticed a remarkably high incidence of postoperative infection (in 6/29 patients) after Hagie pin osteosynthesis, and also osteomyelitis in 2 patients. In the study by Ferran et al. (2010), incidence of postoperative infection in the plate group (3/15) was above the average in the literature. The amount of reported complications varied greatly between the studies. For operative treatment the range was 1.5–76% and for nonoperative treatment it was 1.3–93%.

**Table 4.** Reported complications in the studies (n=13) approved to the systematic review of clavicle fractures

Author, year, country	Methods**^a^**	Non-union	Mal-union	Delayed union	Wound infection	Hardwareorbandage irritation	Mechanical failure of osteosynthesis	Protrusion or dislocation of implant	CRPS**^a^**	Brachial plexus symptoms	Other	All complications (%)
Hoofwijk 1988 Netherlands	RSB M	4/74 0/78		1/74 0/78						0/74 1/78		5/74 (7) 1/78 (1)
COTS**^a^** 2007 Canada	Plate Sling	2/62 7/49	0/62 9/49		3/62	5/62	1/62		0/62 1/49	8/62 7/49	4/62 7/49	23/62 (37) 31/49 (63)
Shen 2008 China	3DPSP			1/67 8/66								1/67 (1) 8/66 (12)
Judd 2009 United States	H pin Sling	1/29 1/28		1/29	6/29 superf. 2/29 deep	9/29	1/29				2/29 1/28	22/29 (76) 2/28 (7)
Smekal 2009, 2011, Austria **^b^**	ESIN Sling	0/60 6/52	0/60 2/52	2/60 9/52	1/60	5/60	9/60			0/60 3/52	1/60 0/52	18/60 (30) 20/52 (39)
Ferran 2010 UK	RP Plate	0/17 0/15			0/17 3/15	1/17 0/15	1/17 0/15				2/17 1/15	4/17 (24) 4/15 (27)
Jubel 2005 Germany	ESIN RSB	0/26 2/27			0/26 0/27	7/26 9/27		4/26 0/27		1/26 4/27	2/26 6/27	14/26 (54) 21/27 (78)
Lee 2007 Taiwan	KP Plate	0/32 1/30			0/32 1/30	4/32 12/30	0/32 2/30					4/32 (13) 16/30 (53)
Lee 2008 Taiwan	KP Plate	0/56 1/32			0/56 1/32	4/56 12/32	0/56 1/32					4/56 (7) 15/32 (47)
Pai 2009 Taiwan	LCP NLP	1/29 1/35			0/29 1/35	11/29 14/35	0/29 4/35					12/29 (41) 20/35 (57)
Hsu 2010 Taiwan	HP TB	0/35 0/30					9/35 0/30	0/35 5/30				9/35 (26) 5/30 (12)
Böhme 2011 Germany	RSB Plate ESIN	1/47 0/53 0/20	1/47 0/53 0/20	1/47 0/53 0/20	2/53 0/20		0/47 4/53 1/20			0/47 0/53 1/20	4/47 1/53 1/20	7/47 (15) 7/53 (13) 3/20 (15)
Kulshrestha 2011, India	Plate Sling	0/45 8/28	2/45 10/28	2/45 0/28		4/45	2/45				2/45 8/28	12/45 (27) 26/28 (93)

**^a^** Abbreviations: CRPS= Complex regional pain syndrome RSB=Rucksack bandage M = Mitella COTS = Canadian Orthopaedic Trauma Society 3DP = Three-dimensional plate SP = Superior plate H pin = Hagie pin ESIN = Elastic stable intramedullary nail RP = Rockwood pin KP = Knowles pin LCP = Locking compression plate NLP = Non-locking plate HP = Hook plate TB = Tension band

**^b^** Studies are assessed to originate from the same patient population. Results are reported from the recent (2011) study.

### Synthesis of evidence

We classified pain, function, and disability as having critical clinical relevance. Delayed union, nonunion, and complications were classified as being important in clinical relevance. No publication bias appeared in the studies included.

*Operative vs. nonoperative treatment.* Data were extracted from 3 randomized controlled trials (COTS 2007, Judd et al. 2009, Smekal et al. 2011) and 3 controlled clinical trials (Jubel et al. 2005, Bohme et al. 2011, Kulshrestha et al. 2011). In favor of surgery, there was very limited evidence (level D) of considerable effectiveness of pain relief at 1–5 months and of low effectiveness (level D) at 6–7 months. Using function (Constant score), moderate-quality evidence (level B) of considerable effectiveness at 6 weeks and of low effectiveness (level B) after the 6-month follow-up was found in favor of surgery. Moderate-quality evidence (level B) of considerable effectiveness in favor of surgery using disability (DASH, L’Insalata) was found at 6 weeks and of low effectiveness (level B) after the 6-month follow-up. Moderate-quality evidence (level B) of similar risk of relatively mild complications was found in patients treated operatively or nonoperatively. There was moderate-quality evidence (level B) of delayed union and nonunion being more common in patients treated nonoperatively than operatively.

*Operative vs. operative treatment.* Evidence was extracted from 2 randomized controlled trials (Shen et al. 2008, Ferran et al. 2010) and 3 controlled clinical trials (Lee et al. 2007, 2008, Pai et al. 2009). There was very limited evidence (level D) of no difference in postoperative pain at 3 days between locking plate and nonlocking plate in elderly patients. Limited evidence (level C) was found of no difference in function (Constant score) at 1 year and later between pin and plate, or locking plate and nonlocking plate, in elderly patients. There was very limited evidence (level D) of smaller need for reoperations in elderly patients initially treated with locking plate than among those treated with nonlocking plate. Limited evidence (level C) of no difference in complications was found in treatment with pin or plate osteosynthesis. There was moderate evidence (level B) that osteosynthesis method has no effect on incidence of delayed union or nonunion.

*Nonoperative vs. nonoperative treatment.* Evidence was extracted from 1 randomized controlled trial (Hoofwijk and van der Werken 1988). There was limited evidence (level C) of no difference in pain between rucksack bandage and mitella at 2 weeks and 6 months.

### Treatment of lateral and medial clavicle fractures

No controlled trials were found on medial clavicle fractures. We found 1 controlled clinical trial on lateral clavicle fractures, comparing hook plate to tension band wire (Hsu et al. 2010). In this study, average time for union was similar between the groups. When comparing range of movement and function between the hook plate group and the tension band group, elevation was 160° vs. 165°, abduction was 165° vs. 168°, and Oxford shoulder score 18 vs. 21.

No major complications appeared in this study. Complications consisted of implant-related subacromial erosion in the hook plate group and K-wire migration in the tension band group.

According to this single study, there was very limited evidence (level D) of no difference in function (ROM, Oxford shoulder score) between hook plate and tension band wire at the 6-month follow-up. There was also very limited evidence (level D) that the osteosynthesis method (hook plate or tension band wire) has no effect on incidence of delayed union or nonunion, and very limited evidence (level D) of no difference in complications between patients treated with hook plate or tension band wire.

## Discussion

We found 6 randomized controlled trials and 7 controlled clinical trials on clavicle fractures published between 1966 until the end of March, 2011. Considering the relevance of the topic, surprisingly few trials have been published, especially on medial and lateral fractures. None of the studies analyzed the correlation between union or nonunion and functional results. Overall, the evidence was mainly graded as limited (level C) or very limited (level D). The moderate-quality evidence (level B) can be summarized as follows: (1) The operative treatment of middle-third clavicle fractures has considerable effectiveness on better function, particularly after short-term follow-up; (2) The operative treatment of middle-third clavicle fractures has considerable effectiveness on less disability, particularly after short-term follow-up; (3) There was similar risk of relatively mild complications after nonoperative or operative treatment of middle-third clavicle fractures; (4) Delayed union and nonunion were more common in patients treated nonoperatively than in those treated operatively; (5) Osteosynthesis method had no effect on the incidence of delayed union or nonunion.

There was only 1 study on lateral clavicle fractures, and it was graded as having very limited (level D) evidence. No studies were found on medial clavicle fractures. There were no major or perilous complications, and evidently some complications were related to a particular treatment option. No clear conclusion can be drawn from the incidence of complications due to the highly heterogeneous reporting.

Some systematic reviews of clavicle fractures have been published earlier. In a review of 2,144 midshaft clavicle fractures, Zlowodzki et al. (2005) reported a 4% nonunion rate in total. With nonoperative treatment, the nonunion rate was 6% for all fractures and 15% for displaced fractures. When treated nonoperatively, fracture displacement, fracture comminution, female gender, and ageing were associated with nonunion. That particular review found plating to be more successful in fracture consolidation than nonoperative treatment, while the results were contradictory when comparing intramedullary fixation and nonoperative treatment. The review included 3 randomized controlled trials with methodological limitations, and also retrospective cohort studies and case series. There was only 1 study comparing different operative methods. Zlowodzki et al. reported only nonunion, infection, and fixation failures while functional outcome measures were not analyzed.

Two Cochrane reviews on clavicle fractures have been published. Lenza et al. (2009a) analyzed 3 studies comparing nonoperative treatments for middle-third clavicle fractures, but they were not able to evaluate the effectiveness of different treatment options. Lenza et al. (2009b) also analyzed studies comparing different operative methods of acute middle-third fractures or nonunion. The review included 3 studies and concluded that there is limited evidence regarding the effectiveness of different operative methods.

*Limitations.* The present work has some limitations. Despite the extensive study search, it is possible that we did not find all the eligible trials. We excluded 3 studies because of a foreign language, and thus may have been missed some information. The quality of the studies varied. There were 2 studies with high risk of bias. Major shortcomings were included, such as improper method of randomization and concealment of allocation, analysis not based on the intention-to-treat principle, and especially unsystematic and insufficient reporting of studies. Due to the clinical heterogeneity (patients, interventions, outcome measures, morphology, and displacement of fractures) we could not perform a meta-analysis. In addition, we were unable to calculate the number–needed-to-treat figures for the trials included, as there were no data for assessment of the minimal clinically important differences or patient-acceptable symptom states (Kvien et al. 2007).

*Implications for practice.* There is moderate-quality evidence that operative treatment of middle-third clavicle fractures leads to slightly better functional results than nonoperative treatment, particularly after short-term follow-up. Also, fracture union was better achieved with surgery. After 6 months, the benefits of operative treatment were very small, as most of the patients also recovered with nonoperative treatment. The studies provide evidence that nonoperative treatment of middle-third clavicle fractures usually leads to adequate functional results, pain relief, and union rates. Operative treatment should be considered to achieve the union for active patients who need to recover to the previous level of activity in the shortest possible time.

*Implications for research.* High-quality, randomized controlled trials comparing plate osteosynthesis, intramedullary nailing, and nonoperative treatment are needed. In particular, randomized controlled trials of lateral and medial clavicle fractures are required. For the moment, is impossible to draw any conclusions regarding treatment of these fractures. More data are required to assess the effectiveness of locking plates and pre-contoured plates in middle and lateral clavicle fractures. Future studies should also assess the impact of fracture union or nonunion on functional outcomes and determine whether to treat operatively only those patients with symptomatic nonunion.

## Supplementary data

Appendix and Table 3 are available on our website (www.actaorthop.org), identification number 4638.
