# Supplementary material for: Operative and nonoperative treatment of clavicle fractures in adults: A systematic review of 1,190 patients from the literature
Source: Acta Orthop. 2012 Feb 8;83(1):65–73. doi: 10.3109/17453674.2011.652884 (PMC3278660; doi:10.3109/17453674.2011.652884)
Supplement: Supplementary file 1 [file ORT-1745-3674-83-065-s4638.pdf]

Supplementary article data

Operative and nonoperative treatment of clavicle fractures in adults

A systematic review of 1,190 patients from the literature

Kaisa J Virtanen[1], Antti O V Malmivaara[2], Ville M Remes[1], and Mika P Paavola[1, 2]

[1]Department of Orthopaedics and Traumatology, Helsinki University Central Hospital; [2]Centre for Health and Social Economics, Institute of Health and Welfare, Helsinki, Finland.
Correspondence: kaisa.virtanen@hus.fi
Submitted 11-01-25. Accepted 11-07-11

Appendix. Information retrieval and strategy to localize candidate trials

1	fractures, bone/ or fractures, closed/ or fractures, comminuted/ or fractures, compression/ or fractures, malunited/ or fractures, open/ or fractures, spontaneous/ or fractures, ununited/ (42617)
2	exp Fracture Fixation/ (31152)
3	Fracture Healing/ (4347)
4	Clavicle/ (2833)
5	(clavic$ or (collar adj2 bone$)).ti,ab. (4029)
6	(fract$ or broken or break$).ti,ab. (451475)
7	1 or 2 or 3 (63904)
8	4 and 7 (963)
9	5 and 6 (1036)
10	8 or 9 (1424)
11	exp Controlled Clinical Trials/ (43438)
12	clinical trial.pt. (421236)
13	((control$ adj5 trial$) or (contr$ adj3 stud$)).ti,ab. (175805)
14	(random$ or rct?).mp. [mp=title, original title, abstract, name of substance word, subject heading word] (438045)
15	control groups/ or cross-over studies/ or double-blind method/ or single-blind method/ (103564)
16	11 or 12 or 13 or 14 or 15 (740095)
17	10 and 16 (48)
18	(consensus or guideline$).mp. (168501)
19	National Health Programs/ (14023)
20	18 or 19 (181713)
21	10 and 20 (8)
22	exp Longitudinal Studies/ (512951)
23	(follow up or follow?up or prospect$).ti,ab. (498907)
24	22 or 23 (753141)
25	10 and 24 (264)
26	(systemat$ adj2 review$).ti,ab. (9244)
27	meta-analysis/ (6422)
28	meta-analy$.ti,ab. (14040)
29	meta-analysis.pt. (12027)
30	metaregressi$.mp. [mp=title, original title, abstract, name of substance word, subject heading word] (43)
31	review.pt. (1157935)
32	evidence-based medicine/ (19222)
33	26 or 27 or 28 or 29 or 30 or 31 or 32 (1186377)
34	10 and 33 (110)
35	17 or 21 or 25 or 34 (400)
36	from 35 keep 1-200 (200)

Open Access - This article is distributed under the terms of the Creative Commons Attribution Noncommercial License which permits any noncommercial use, distribution, and reproduction in any medium, provided the source is credited.
DOI 10.3109/17453674.2011.652884

Kaisa J Virtanen

Table 3. Effectiveness of the included studies (n=13) in the systematic review of clavicle fractures.

Author, year, country	Primary outcome measure	Secondary outcome measures	Complications	Union	Nonunion
**Hoofwijk 1988**<br>**Netherlands**	**Pain**: VAS [(a)] (p>0.05)<br>Rucksack bandage:<br>1. day: 5.3<br>8. day: 3.3<br>15. day: 2.6<br>Mitella:<br>1. day: 5.3<br>8. day: 3.0<br>15. day: 1.8	**Need for analgesic medication** (p>0.05)<br>Rucksack bandage: 4.2 days<br>Mitella: 3.6 days<br>**Return to work** (p>0.05)<br>Rucksack bandage: 3.6 weeks<br>Mitella: 3.5 weeks<br>**Return to sports** (p>0.05)<br>Rucksack bandage: 5.0 weeks<br>Mitella: 5.6 weeks<br>**Union**: clinical evaluation (p>0.05)<br>Rucksack bandage: 3.8 weeks<br>Mitella: 3.6 weeks<br>**Function**: mobility of shoulder joint at 10 months (p>0.05)<br>Rucksack bandage: good 73/74<br>Mitella: good 77/78<br>**Muscular strength**<br>Results not reported	**Rucksack bandage**<br>Delayed union: 1<br>Nonunion: 4<br>**Mitella**<br>Secundaric plexus irritation: 1<br>**Total**<br>Rucksack bandage: 5/74<br>Mitella: 1/78	Rucksack bandage: 3.8 weeks<br>Mitella: 3.6 weeks	Rucksack bandage: 4/74<br>Mitella: 0/78
**Canadian Orthopaedic Trauma Society 2007**<br>**Canada**	**Function**: DASH [(1)]<br>at 6 weeks (p<0.01)<br>Plate: 22<br>Sling: 40<br>at 3 months (p=0.04)<br>Plate: 15<br>Sling: 22<br>at 1 year (p<0.01)<br>Plate: 5<br>Sling: 15	**Function**: CS [(1)]<br>at 6 weeks (p<0.01)<br>Plate: 80<br>Sling: 69<br>at 3 months (p<0.01)<br>Plate: 90<br>Sling: 83<br>at 1 year (p<0.01)<br>Plate: 96<br>Sling: 90<br>**Union**: X-ray (p=0.001)<br>Plate: 16.4 weeks<br>Sling: 28.4 weeks<br>**Complications**: (p=0.008)<br>Plate: 23/62<br>Sling: 31/49	**Plate**<br>Nonunion: 2<br>Wound infection: 3<br>Hardware irritation requiring removal: 5<br>Transient brachial plexus symptoms: 8<br>Abnormality in AC/STC joint: 2<br>Early mechanical failure: 1<br>Other: 2<br>**Sling**<br>Nonunion: 7<br>Malunion requiring treatment: 9<br>CRPS: 1<br>Surgery for impending open fracture: 2<br>Transient brachial plexus symptoms: 7<br>Abnormality in AC/STC joint: 3<br>Other: 2<br>**Total** (p=0.008)<br>Plate: 23/62<br>Sling: 31/49	Plate: 16.4 weeks<br>Sling: 28.4 weeks<br>(p=0.001)	Plate: 2/62<br>Sling: 7/49<br>(p=0.042)
**Shen 2008**<br>**China**	**Union:** X-ray at 4 months [(a)]<br>3D plate: 63/67<br>Superior plate: 43/66	**ROM:** results not reported<br>**Strength:** results not reported<br>**Symptoms beyond 16 weeks** (p=0.015)<br>3D plate: 3/66	**3D plate**<br>Delayed union: 1<br>**Superior plate**<br>Delayed union: 8	3D plate: 66/66<br>Superior plate: 58/58	3D plate: 0/66<br>Superior plate: 0/58

Kaisa J Virtanen

		Superior plate: 15/58<br>**Delayed union** X-ray at 4 months (p=0.018)<br>3D plate: 1/66<br>Superior plate: 8/58<br>**Operation time** (p=0.334): results not reported<br>**Hospital stay** (p=0.361): results not reported	**Total**<br>3D plate: 1/66<br>Superior plate: 8/58		
**Judd 2009**<br>**United States**	**Function:** SANE [(a)]<br>at 6 weeks (p>0.05)<br>Hagie pin: 65.9<br>Sling: 56.1<br>at 3 months (p>0.05)<br>Hagie pin: 78.5<br>Sling: 70.7<br>at 1 year (p>0.05)<br>Hagie pin: 93.5<br>Sling: 97.0	**Function:** L'Insalata<br>at 6 weeks (p>0.05)<br>Hagie pin: 61.3<br>Sling: 51.6<br>at 3 months (p>0.05)<br>Hagie pin: 73.5<br>Sling: 66.4<br>at 1 year (p>0.05)<br>Hagie pin: 95.5<br>Sling: 97.9<br>**Union:** X-ray (p>0.05)<br>Hagie pin: 26/29<br>Sling: 22/28	**Hagie pin**<br>Prominent pin head: 9<br>Superficial pin-tract infection: 6<br>Osteomyelitis: 2<br>Transient radial nerve palsy: 1<br>Delayed union: 1<br>Nonunion: 1<br>Pin breakage: 1<br>Refracture: 1<br>**Sling**<br>Nonunion: 1<br>Refracture: 1<br>**Total**<br>Hagie pin: 22/29<br>Sling: 2/28	Hagie pin: 28/29<br>Sling: 27/28	Hagie pin: 1/29<br>Sling:1/28
**Smekal 2009,**<br>**Smekal 2011**<br>**Austria** [(*)]	**Time to union:** X-ray [(a)]<br>(p=0.01)<br>ESIN: 11.9 weeks<br>Sling: 16.6 weeks	**Clavicular shortening**: X-ray<br>ESIN: 3.5% (p<0.05)<br>Sling: 5.1% (p=0.68)<br>**Function:**<br>DASH at 2 years (p=0.03)<br>ESIN: 0.5<br>Sling: 3.1<br>CS at 2 years (p=0.02)<br>ESIN: 98.0<br>Sling: 95.1<br>**Complications** (p=0.55)<br>ESIN: 18/60<br>Sling: 20/52	**ESIN**<br>Delayed union: 2<br>Telescoping: 7<br>Infection: 1<br>Skin irritation: 5<br>Implant failure: 2<br>Refracture: 1<br>**Sling**<br>Nonunion: 6<br>Delayed union: 9<br>Symptomatic malunion: 2<br>Transient plexus irritation: 3<br>**Total** (p=0.55)<br>ESIN: 18/60<br>Sling: 20/52	ESIN: 60/60<br>Sling: 46/52	ESIN: 0/60<br>Sling: 6/52
**Ferran 2010**<br>**United Kingdom**	**Function:** CS at 1 year<br>(p=0.365)<br>Rockwood pin: 92.1<br>Plate: 88.7	**Function:** Oxford shoulder score at 1 year<br>(p=0.773)<br>Rockwood pin: 45.2<br>Plate: 44.7<br>**Union**: X-ray<br>Rockwood pin: 17/17<br>Plate: 15/15<br>**Complications**:<br>Rockwood pin: 4/17<br>Plate: 4/15	**Rockwood pin**<br>Soft tissue irritation: 1<br>Scar numbness: 2<br>Metalwork loosening: 1<br>**Plate**<br>Scar numbness: 1<br>Superficial wound infection: 3<br>**Total**<br>Rockwood pin: 4/17<br>Plate: 4/15	Rockwood pin: 17/17<br>Plate: 15/15	Rockwood pin: 0/17<br>Plate: 0/15

**Jubel 2005**<br>**Germany**	**Pain**: VAS [a] [1]<br>at 3 days (p<0.05)<br>ESIN: 31<br>Rucksack bandage: 72<br>at 7 days (p<0.05)<br>ESIN: 28<br>Rucksack bandage: 63<br>at 2 weeks (p<0.05)<br>ESIN: 15<br>Rucksack bandage: 52<br>at 3 weeks (p<0.05)<br>ESIN: 15<br>Rucksack bandage: 45<br>at 4 weeks (p<0.05)<br>ESIN: 8<br>Rucksack bandage: 29<br>at 3 months (p<0.05)<br>ESIN: 4<br>Rucksack bandage: 15<br>at 6 months (p<0.05)<br>ESIN: 1<br>Rucksack bandage: 14	**Satisfaction**: 1-6 [1] (p<0.001)<br>ESIN: 1.1<br>Rucksack bandage: 2.0<br>**Duration of sick leave**: days (p<0.001)<br>ESIN: 16 ±12<br>Rucksack bandage: 40 ±19<br>**Function**: CS at 6 months [1] (p<0.001)<br>ESIN: 98<br>Rucksack bandage: 90<br>**Shortening of clavicle**: cm (p<0.001)<br>ESIN: 0.2 ±0.3<br>Rucksack bandage: 1.4 ±0.9<br>**ROM**: Abduction at 6 months [1] (p<0.05)<br>ESIN: 175°<br>Rucksack bandage:165°<br>**Function:** DASH at 6 months [1] (p<0.001)<br>ESIN: 2<br>Rucksack bandage: 10<br>**Complications**<br>ESIN: 14/26<br>Rucksack bandage: 21/27	**ESIN**<br>Paresthesia of ipsilatereral arm: 1<br>Pain in the medial end of nail: 7<br>Lateral peforation of nail: 2<br>Shortening of nail tip: 2<br>Early removal of nail: 2<br>**Rucksack bandage**<br>Paresthesia of ipsilatereral arm: 4<br>Painfull skin lesion in axilla: 9<br>Swelling and syanose of ipsilateral arm: 4<br>Loss of reposition: 2<br>Nonunion: 2<br>**Total**<br>ESIN: 14/26<br>Rucksack bandage: 21/27	ESIN: 26/26<br>Rucksack bandage: 25/27	ESIN: 0/26<br>Rucksack bandage: 2/27
**Lee 2007**<br>**Taiwan**	**Union:** X-ray at 6 months [a] (p=0.48)<br>Knowles pin: 32/32<br>Plate: 29/30	**Function:** CS at 30 months (p=0.7)<br>Knowles pin: 85<br>Plate: 84<br>**Operative time:** minutes (p<0.001)<br>Knowles pin: 36<br>Plate: 64<br>**Incision length:** cm (p=0.001)<br>Knowles pin: 4.2<br>Plate: 7.8<br>**Hospital stay:** days (p=0.03)<br>Knowles pin: 6.2<br>Plate: 9.1	**Knowles pin**<br>Hardware irritation: 4<br>**Plate**<br>Nonunion: 1<br>Wound infection: 1<br>Implant failures: 2<br>Hardware irritation: 12<br>**Total** (p=0.04)<br>Knowles pin: 4/32<br>Plate: 16/30	Knowles pin: 32/32<br>Plate: 29/30	Knowles pin: 0/32<br>Plate: 1/30<br>(p=0.48)
**Lee 2008**<br>**Taiwan**	**Union:** X-ray at 6 months [a] (p=0.36)<br>Knowles pin: 56/56<br>Plate: 31/32	**Operative time:** minutes (p<0.001)<br>Knowles pin: 27.5<br>Plate: 68.4<br>**Pain:** VAS<br>Results not reported<br>**Function:** CS (p=0.84)<br>Knowles pin: 95.3<br>Plate: 93.1<br>**Incision length:** cm (p<0.001)<br>Knowles pin: 4.1<br>Plate: 8.4	**Knowles pin**<br>Hardware irritation: 4<br>**Plate**<br>Nonunion: 1<br>Hardware irritation: 12<br>Wound infection: 1<br>Implant failure: 1<br>**Total** (p=0.045)<br>Knowles pin: 4/56<br>Plate: 15/32	Knowles pin: 56/56<br>Plate: 31/32<br>(p=0.36)	Knowles pin: 0/56<br>Plate: 1/32

Kaisa J Virtanen

**Pai 2009**<br>**Taiwan**	**Union:** X-ray at 6 months [(a)]<br>(p=1.0)<br>LCP: 28/29<br>NLP: 34/35	**Pain:** VAS preop. - 3. day postop. (p=0.91)<br>LCP: 6.7 - 2.1<br>NLP: 6.5 - 2.2<br>**Function:** CS at 1 year (p=0.54)<br>LCP: 91.6<br>NLP: 89.1	**LCP**<br>Nonunion: 1<br>Symptomatic hardware: 11<br>**NLP**<br>Nonunion: 1<br>Plate loosening: 4<br>Wound infection: 1<br>Symptomatic hardware: 14<br>**Total** (p=0.087)<br>LCP:12/29<br>NLP: 20/35	LCP: 28/29<br>NLP: 34/35<br>(p=1.0)	LCP: 1/29<br>NLP: 1/35
**Hsu 2010**<br>**Taiwan**	**Union:** X-ray [(a)]<br>Hook plate: 14.2 weeks<br>Tension band: 13.8 weeks	**Function:** ROM at 6 months<br>Elevation (p=0.006)<br>Hook plate: 160°<br>Tension band: 165°<br>Abduction (p=0.004)<br>Hook plate: 165°<br>Tension band: 168°<br>External rotation (p=0.396)<br>Hook plate: 80° 4/35, 85° 18/35, 90° 13/35<br>Tension band: 85° 18/30, 90° 11/30, 95° 1/30<br>**Function:** Oxford shoulder score at 6 months (p=0.072)<br>Hook plate: 21<br>Tension band: 18	**Hook plate**<br>Subacromial erosion: 9<br>**Tension band**<br>K-wire migration: 5<br>**Total**<br>Hook plate: 9/35<br>Tension band: 5/30	Hook plate: 14.2 weeks<br>Tension band: 13.8 weeks	Hook plate: 0/35<br>Tension band: 0/30
**Böhme 2011**<br>**Germany**	**Pain:** VAS (0-15) [(a)]<br>(p=0.316)<br>Results not reported	**Cosmetics** (p=0.48)<br>Results not reported<br>**Union**<br>Results not reported<br>**Function**: CS at 8 months (p=0.01)<br>Rucksack bandage: 90.3<br>Plate: 94.4<br>ESIN: 96.7<br>**Complications**<br>Rucksack bandage: 7/47<br>Plate: 7/53<br>ESIN: 3/20	**Rucksack bandage**<br>Deep vein thrombosis: 1<br>Malunion: 1<br>Skin tightening: 1<br>Nonunion: 1<br>Intolerable pain: 2<br>Delayed union: 1<br>**Plate**<br>Wound healing problem: 1<br>Wound infection: 2<br>Plate bending: 3<br>Plate breakage: 1<br>**ESIN**<br>Plexus irritation: 1<br>Wound healing problem: 1<br>Nail breakage: 1<br>**Total**<br>Rucksack bandage: 7/47<br>Plate: 7/53<br>ESIN: 3/20	Rucksack bandage: 46/47<br>Plate: 53/53<br>ESIN: 20/20	Rucksack bandage: 1/47<br>Plate: 0/53<br>ESIN: 0/20
**Kulshrestha 2011**	**Union:** X-ray at 6 months	**Function**: CS at 18 months (p<0.0001)	**Plate**	Plate: 45/45	Plate: 0/45

Kaisa J Virtanen

**India**	(p=0.002)<br>Plate: 45/45<br>Sling: 20/28	Only difference (5.8) between groups reported<br>**Complications**<br>Plate: 12/45<br>Sling: 26/28	Implant failure: 2<br>Delayed union: 2<br>Reoperation: 2<br>Symptomatic malunion: 2<br>Implant irritation and removal: 4<br>**Sling**<br>Nonunion: 8<br>Symptomatic malunion: 10<br>Surgery for nonunion/malunion: 8<br>**Total**<br>Plate: 12/45<br>Sling: 26/28	Sling: 20/28	Sling: 8/28

(a) Primary outcome was not specified in the study. Selected primary outcome is the first mentioned in Materials and Methods –section.
If outcomes were not mentioned in Materials and Methods – section, the selected primary outcome is the first mentioned in Results-section.

(1) Values estimated from diagram.

(*) Studies are assessed to originate partly from the same patient population. Results are from the recent (2011) study.

Abbreviations:

VAS= Visual Analogue Scale
DASH= Disabilities of the Arm, Shoulder and Hand score
CS= Constant Shoulder Score
AC= Acromioclavicular
STC= Sternoclavicular
CRPS= Complex Regional Pain Syndrome
3D= Three-dimensional
SANE= Single Assessment Numeric Evaluation
ESIN= Elastic Stable Intramedullary Nail
ROM= Range Of Motion
LCP= Locking Compression Plate
NLP= Non-Locking Plate
